# Efficacy of Nondiuretic Pharmacotherapy for Improving the Treatment of Congestion in Patients with Acute Heart Failure: A Systematic Review of Randomised Controlled Trials

**DOI:** 10.3390/jcm11113112

**Published:** 2022-05-31

**Authors:** Abdelrahman N. Emara, Noha O. Mansour, Mohamed Hassan Elnaem, Moheb Wadie, Inderpal Singh Dehele, Mohamed E. E. Shams

**Affiliations:** 1 Clinical Pharmacy and Pharmacy Practice Department, Faculty of Pharmacy, Mansoura University, Mansoura 35516, Egypt; a.emaraa@mans.edu.eg (A.N.E.); nohamansaur@mans.edu.eg (N.O.M.); mshamspharma@gmail.com (M.E.E.S.); 2 Department of Pharmacy Practice, Faculty of Pharmacy, International Islamic University Malaysia, Kuantan 25200, Pahang, Malaysia; 3 Quality Use of Medicines Research Group, Faculty of Pharmacy, International Islamic University Malaysia, Kuantan 25200, Pahang, Malaysia; 4 Cardiology Department, Faculty of Medicine, Mansoura University, Mansoura 35516, Egypt; muheb2001@hotmail.com; 5 School of Pharmacy, University of Birmingham, Birmingham B15 2TT, UK; i.s.dehele@bham.ac.uk

**Keywords:** acute heart failure, decongestion, decompensation, dyspnea, adjuvant, tolvaptan, serelaxin, acute decompensated heart failure, levosimendan, empagliflozin

## Abstract

Diuretic therapy is the mainstay during episodes of acute heart failure (AHF). Diuretic resistance is often encountered and poses a substantial challenge for clinicians. There is a lack of evidence on the optimal strategies to tackle this problem. This review aimed to compare the outcomes associated with congestion management based on a strategy of pharmacological nondiuretic-based regimens. The PubMed, Cochrane Library, Scopus, and ScienceDirect databases were systematically searched for all randomised controlled trials (RCTs) of adjuvant pharmacological treatments used during hospitalisation episodes of AHF patients. Congestion relief constitutes the main target in AHF; hence, only studies with efficacy indicators related to decongestion enhancement were included. The Cochrane risk-of-bias tool was used to evaluate the methodological quality of the included RCTs. Twenty-three studies were included; dyspnea relief constituted the critical efficacy endpoint in most included studies. However, substantial variations in dyspnea measurement were found. Tolvaptan and serelaxin were found to be promising options that might improve decongestion in AHF patients. However, further high-quality RCTs using a standardised approach to diuretic management, including dosing and monitoring strategies, are crucial to provide new insights and recommendations for managing heart failure in acute settings.

## 1. Introduction

Acute heart failure (AHF) is a life-threatening clinical syndrome requiring urgent hospitalisation [1]. Dyspnea resulting from excessive fluid retention is the most common presenting symptom among hospitalised patients. The initial treatment goal is to achieve decongestion without residual fluid retention [2]. Although management options for chronic heart failure continue to expand, similar advances have not been achieved in acute settings. Cardiac transplantation is the treatment of choice for patients with end-stage heart failure who remain symptomatic despite optimal medical therapy. For carefully selected patients, heart transplantation offers improved survival and quality of life [3,4].

Acute heart failure management is associated with 4 to 10% in-hospital mortality, and post-discharge 1-year mortality is up to 25 to 30% [5]. At discharge, inadequate decongestion is a major contributing factor to hospital readmission and poor survival [6]. Currently, decongestive regimens rely mainly on traditional diuretics to combat excessive fluid retention [7]. Diuretic resistance, defined as persistent signs and symptoms despite optimal diuretic dosing, is frequently experienced in AHF patients [8]. This problem is more often encountered in patients with diabetes and/or renal impairment [8,9]. Complex interactions between cardiac and renal dysfunction, renal adaptation, and escape mechanisms such as the braking phenomenon are all thought to play a role in diuretic resistance [7,10,11].

The European society of cardiology’s (ESC) 2021 guidelines recommend the use of IV loop diuretics for all patients with AHF admitted with fluid overload and the addition of a thiazide diuretic when diuresis remains insufficient despite optimal dosing, but these recommendations are based on limited data demonstrating the relative safety of this approach [5]. Notably, the combined diuretics potentially lead to complications such as hyponatremia, hypokalemia, and worsening renal function [10,12,13]. Due to the adverse impact of diuretic combination therapy on electrolyte balance and neurohormonal activation, additional decongestive therapies, such as alternative adjuvant therapies, are urgently needed. Several approaches have been proposed to tackle diuretic resistance. These include exploring the adjuvant use of novel vasodilators and inotropes to the standard diuretic regimens. Furthermore, the recent promising results of using some novel agents in chronic settings, such as sodium-glucose cotransporter-2 inhibitors [14,15,16,17], have encouraged the expansion of the clinical trials to explore their benefits in acute settings [18]. It is critical to improve clinical understanding of the available data to overcome diuretic resistance. Therefore, this review aims to discuss, summarise, and compare the outcomes associated with congestion management in patients with AHF based on a strategy of pharmacological nondiuretic-based regimens.

## 2. Materials and Methods

The findings of this systematic review were reported using the Preferred Reporting Items for Systematic Reviews and Meta-Analyses (PRISMA) guideline.

### 2.1. Data Sources and Search Strategy

**Figure 1:**
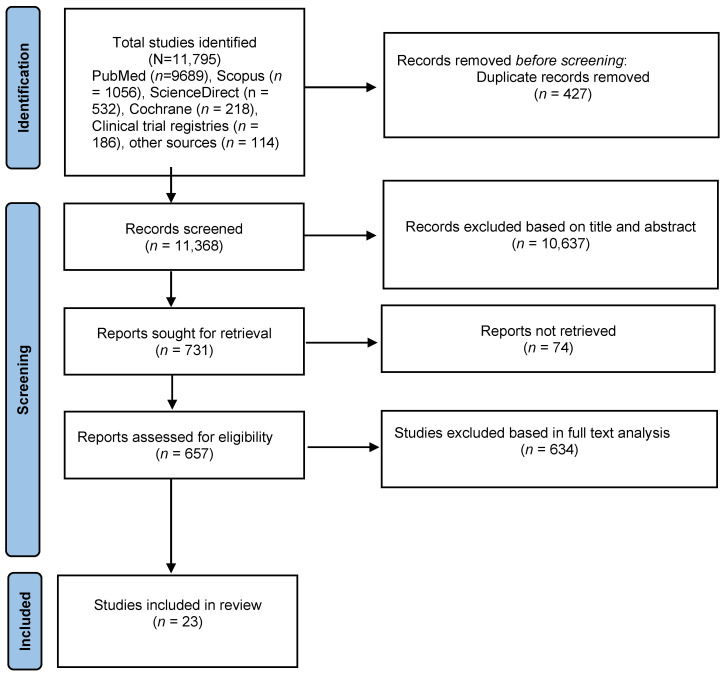
Flow chart of the included studies.

Four scholarly databases, ScienceDirect, Cochrane, Scopus, and PubMed, were systematically searched for clinical trials involving interventions of add-on nondiuretic pharmacotherapy to standard care in AHF treatment up to 30 November 2021. The comparator was either placebo or no drug. Clinical trial registries (ClinicalTrials.gov) were searched for AHF trials associated with the use of adjuvants to diuretics to retrieve relevant studies. The reference lists of recent relevant reviews [19,20,21,22] were searched to ensure literature saturation. The search strategy included MeSH terms and the keywords “acute heart failure” or “cardiac failure” or “acute decompensated heart failure” AND “decongestion” OR “diuresis” OR “dyspnea”. To find the potentially eligible articles, individual names of agents and drug classes in clinical trials were considered. Citation analysis was performed on Google Scholar to track the prospective citing of reference of the included articles. Figure 1 shows the flow chart describing the studies’ selection.

### 2.2. Study Screening and Selection

Original research articles published in English on the use of nondiuretic pharmacotherapy in heart failure patients hospitalised with symptoms of congestion were eligible for inclusion. Two authors independently screened each potentially relevant article’s title, abstract, and full text for inclusion eligibility (A.N.E. and N.O.M.). Any disagreements were resolved by a third author (M.H.E.), and all decisions were made unanimously. For inclusion and assessment of methodological quality, eligible studies were retrieved in full text.

### 2.3. Data Extraction

A standardised form was used to extract data from the selected studies. The following basic information was extracted from the RCTs: the authors, publication date, sample size, patient population, interventions, study design, outcomes, relevant results, and conclusions. Furthermore, the studies were classified pharmacologically, and key findings were reported consistently.

### 2.4. Eligibility Criteria

To determine which RCT to include in the review, we used decongestion efficacy indicators such as dyspnea severity, diuretic response, urine output, and objective decongestion outcomes such as changes in pulmonary capillary wedge pressure (PCWP) or serum natriuretic peptide levels. The exclusion criteria were as follows: (i) retrospective secondary analysis of the RCT, (ii) pilot studies (less than 80 patients), (iii) studies evaluating non-pharmacological drug therapy and/or conventional adjuvant diuretics, (iv) unavailable full texts, (v) editorials, conference abstracts, and short communications.

### 2.5. Quality Assessment

The Cochrane Risk-of-Bias (RoB) tool was used to assess the methodological quality of the included studies [23] independently by two authors. Each item was classified as having low, high, or unclear risk. The randomisation sequence generation, concealment of allocation, blinding of participants and personnel, blinding of outcome assessment, incomplete outcome data, and selective reporting were all used to assess bias in each trial.

## 3. Results

The primary electronic search resulted in the identification of 11,795 studies. We eliminated 427 duplicate studies using EndNote X9 (Thomson Reuters, Toronto, ON, Canada). The remaining 11,368 studies were evaluated for inclusion by determining their relevance. Only 657 of those were determined to be eligible for full-text analysis. Following that, 634 studies were excluded for failing to meet the inclusion criteria. Finally, this review included twenty-three studies that examined the efficacy of a nondiuretic strategy for improving congestion in patients with AHF (Figure 1).

### 3.1. Overview of the Included Studies

Table 1, Table 2 and Table 3 illustrate an overview of the summary of the studies based on trial design, study population, and conclusions. The countries of origin were multinational studies (*n* = 12), the USA (*n* = 5), Japan (*n* = 2), the Netherlands (*n* = 1), and China (*n* = 1). The review included twenty-three RCTs with sample sizes ranging from 80 to 7141 patients. A more detailed summary table of the included studies is provided as a Supplementary File.

### 3.2. Effect of Adjuvant Therapy on Dyspnea

Dyspnea is one of the main reasons for hospitalisation. Our results showed that twelve studies used dyspnea relief as the primary endpoint of the included RCTs, while seven used it as the secondary endpoint. Improvement in dyspnea relief was seen in the experimental group in ten studies. The adjuvant use of tolvaptan [24,27,29] and serelaxin [32,33] have been linked with the most promising and consistent effects. The severity of dyspnea was assessed mainly by patients. Nesiritide has not shown clear improvement in dyspnea. Similarly, the cardiac myosin activator omecamtiv mecarbil and the endothelin receptor inhibitor tezosentan did not relieve dyspnea.

Concerning the assessment procedure, detailed information about supplemental oxygen use or body position during dyspnea assessment was seldomly reported. Furthermore, diverse scales and different timings were common during data extraction. The dyspnea measurement was frequently expressed by Likert scale and/or VAS. Some Dyspnea relief is thought to be due to decongestion, yet it is largely subjective. This variability becomes critical when being assessed in trials across varied geographic regions. Therefore, it is reasonable to explain the failure of clinical trials to inform drug development in AHF patients, at least partly, by the heterogeneity in the endpoints selected to evaluate different interventions.

### 3.3. Effect of Adjuvant Therapies on Natriuretic Peptides

Although the value of natriuretic peptides as surrogate decongestion markers remains controversial [47], it was evaluated as a secondary outcome in four studies and as a co-primary endpoint in one. Overall, changes in natriuretic peptides serum levels were comparable among intervention and control groups with most of the investigated therapeutic options.

### 3.4. Effect of Adjuvant Therapies on Body Weight Change and Urine Output

Bodyweight does not always correlate with intravascular volume, and consequently, its validity as a surrogate marker remains doubtful [47]. The effects of adjuvant drug therapy on this short-term outcome were assessed in four studies. Short-term body weight loss was more pronounced with the add-on vasopressin receptor antagonists, namely tolvaptan. The diuretic response evaluated by the cumulative change in weight (kg) adjusted for the cumulative loop diuretic dose was used in one study [43]. The incremental natriuretic benefits of adding adjuvant therapy to loop diuretics via urine sodium output-based endpoints were not assessed in any of our included studies.

### 3.5. Methodological Quality of Studies

**Figure 2:**
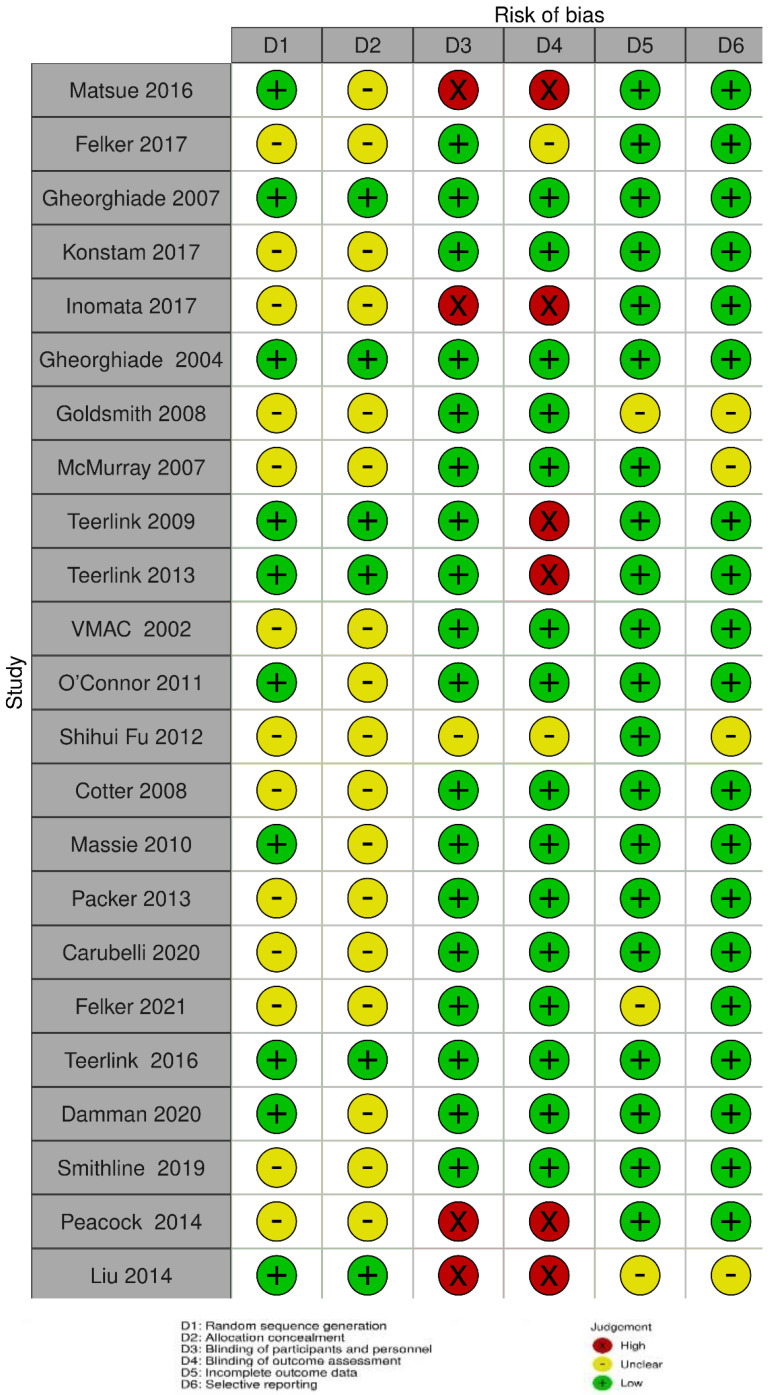
The quality of included RCTs: risk-of-bias per item for each study.

**Figure 3:**
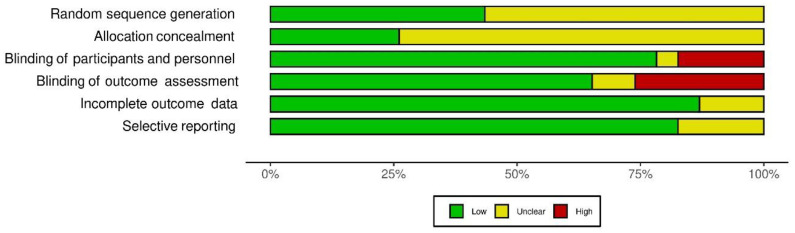
The quality of included RCTs: risk-of-bias per item presented as percentages across all included RCTs.

Figure 2 and Figure 3 show the findings of the critical appraisal of the quality of the included RCTs assessed by the Cochrane RoB assessment tool. Most studies included in this review lacked adequate random sequence generation, and the details of allocation concealment were unclear.

## 4. Discussion

### 4.1. Optimising Vascular Resistance: Novel Vasodilatory Therapies

Apart from diuresis with conventional joint diuretics, the vasodilatory pathway is the most promising therapeutic target in AHF. Fifteen studies have been performed with six different therapeutic options that produce vasodilation as a dominant pharmacological effect to enhance the decongestion of acutely decompensated patients.

#### 4.1.1. Vasopressin Antagonists

Two agents were clinically investigated in acute settings, conivaptan and tolvaptan. Our results included seven reports that evaluated the short-term efficacy of add-on vasopressin antagonists in patients with AHF. Vasopressin antagonists enhanced decongestion efficacy in five studies. These are perhaps explained by the discouragement of arginine vasopressin’s systemic effects, which directly contribute to vasoconstriction and aldosterone release [48]. Although adjuvant tolvaptan has been studied in different doses ranging from (15–90 mg/day) in our included studies, the most substantial evidence emerged from the multinational EVEREST trial [26] (*n* = 4133), which reported favourable outcomes in terms of dyspnea relief with the use of 30 mg/day tolvaptan. Similarly, a recent metanalysis [49] confirmed that add-on tolvaptan effectively relieved dyspnea and decreased body weight in AHF patients.

Traditional diuretics rapidly reduce blood volume and activate the RAAS. Thus, joint use of loop diuretics with the other conventional diuretics might improve decongestion but is typically associated with an increased risk of worsening renal functions (WRF). In contrast, tolvaptan possesses a weaker ability to RAAS activation. This might explain how lower doses of tolvaptan significantly reduced the incidence of WRF in the subgroup analysis in the previously mentioned meta-analysis [49]. However, opposite actions were reported with the higher doses of tolvaptan (30 mg/day); it has been correlated with an increased risk of WRF. These differential effects need further investigations to be justified. So far, the current evidence suggests that tolvaptan effectively improves decongestion in patients with AHF, particularly with high doses. However, the increased risk of WRF could not be dismissed. Furthermore, the high cost of tolvaptan may also limit its clinical utility [21].

#### 4.1.2. Serelaxin

A recombinant human relaxin-2, serelaxin is a vasodilator agent with end-organ protective anti-inflammatory effects [22]. It exerts its effects through cyclic AMP and activation of the endothelin type B receptor [48]. Serelaxin yielded promising results in the Pre-RELAX-AHF preliminary study. The larger (RELAX-AHF) trial (*n* = 1161 patients) confirmed thereafter the clinical utility of serelaxin as it markedly improved dyspnea relief. A recent meta-analysis confirmed that serelaxin was associated with favourable outcomes in reducing the incidence of WHF [50]. The diversity of the methods used to assess dyspnea has precluded performing a meta-analysis of the effects of serelaxin on dyspnea. However, serelaxin significantly improved biomarkers of congestion, during the first 48 h [50], according to the analysis results. The safety profile and the available evidence suggest that treatment with serelaxin relieves dyspnea, yet, it has not been approved in AHF patients. This might be illustrated by the results of the RELAX-AHF-2 trial [51], designed to evaluate the effects of serelaxin on post-discharge mortality and WHF and the pragmatic multicenter RELAX-AHF-EU trial [52] that was designed to assess the effect of serelaxin on in-hospital WHF. Despite a trend towards less in-hospital HF worsening with serelaxin, RELAX-AHF-2 failed to meet the co-primary endpoint. Similarly, RELAX-AHF-EU was terminated early. The international large trial results are neutral, leading to no possibility of regulatory approval for the commercialisation of serelaxin [53].

#### 4.1.3. Rolofylline

Rolofylline is an adenosine A_1_ antagonist that blocks receptors in the renal afferent arterioles. Increased adenosine concentration has been associated with diuretic resistance [54]. The most substantial evidence of the value of rolofylline use emerged from PROTECT [38], which failed to show clinically meaningful symptom improvement with its adjuvant use. Differences in the inclusion criteria and sample size explain the lack of consistency between the PROTECT [38] results and the preliminary results of the dose-finding study [37]. Considering the lack of proven efficacy, the complications of adenosine receptor antagonists, and the results that emerged from the safety analysis of PROTECT [55], the rolofylline development program has been terminated [54].

#### 4.1.4. Nesiritide

Nesiritide is an exogenous recombinant BNP that increases vasodilation and augments natriuresis in patients with ADHF. Promising results were initially seen in VMAC [34]. Nesiritide was associated with a more significant dyspnea improvement when compared to placebo. Contrarily, similar findings have not been replicated in larger studies. Concerning the (ASCEND-HF) trial [35], nesiritide did not improve decongestion in AHF patients. Dopamine is an endogenous catecholamine that, at low doses (≤3 μg/kg/min), may selectively activate dopamine receptors and promote renal vasodilatation. Previous studies have suggested that the addition of low dose dopamine to diuretic therapy enhances decongestion and preserves renal function during diuretic therapy in acute heart failure [56,57]. In line with ASCEND-HF results, the ROSE-AHF trial [58] evaluated the efficacy of nesiritide versus dopamine; nesiritide did not enhance fluid removal when added to loop diuretic therapy. So, nesiritide is no longer used in the treatment of HF.

#### 4.1.5. Tezosentan

Tezosentan, a non-selective antagonist of the endothelin 1 receptor, reduces LV filling pressure, systemic vascular resistance, and plasma BNP levels [54]. The value of its use in AHF was evaluated in one study (VERITAS) [31], which was prematurely stopped because of a low probability of achieving a significant treatment effect. Tesozentan did not affect dyspnea or the rate of WRF when compared to placebo. As a result, it is not currently used in AHF treatment.

### 4.2. Optimising Inotropy

The administration of traditional inotropic agents is associated with an increase in ventricular tachyarrhythmias, atrial fibrillation, and myocardial ischemia. Based on safety concerns of increased mortality, the latest recommendation of the ESC limited the use of inotropes only to patients with SBP < 90 mmHg and evidence of hypoperfusion who do not respond to standard treatment [5].

#### 4.2.1. Optimising Inotropy: Novel Calcitrope Therapies

##### Levosimendan

Levosimendan has three major mechanisms of action: positive inotropy, vasodilation, and cardiac cytoprotection [59]. In the REVIVE I and II trials, the primary endpoint of change in clinical course showed more significant improvements in the levosimendan group compared with the placebo group. However, hypotension and atrial fibrillation occurred more often in the levosimendan group. Additionally, levosimendan increased early mortality [39]. Post hoc analyses of the dataset found baseline SBP < 100 mmHg or DBP < 60 mmHg as a factor associated with increased mortality risk. However, a re-analysis of the mortality data excluding patients with hypotension eliminated the excess mortality in the levosimendan cohort [60]. Moreover, a meta-analysis of 5349 patients concluded that levosimendan therapy increased the risk of recurrence of extrasystoles in patients with AHF [61]. On the other hand, some studies have demonstrated levosimendan’s benefits compared to other inotropes, especially those with adrenergic mechanisms, while others have bordered on detrimental results with levosimendan [22]. Thus, the utility of levosimendan in managing AHF is now controversial, particularly in patients with hypotension. In line with these findings, excessive peripheral vasodilation and hypotension were recently highlighted in the updated guidelines as significant levosimendan limitations, especially when administered at high doses and/or when commenced with a bolus dose [5].

##### Istaroxime

The use of istaroxime has been reported only in one clinical trial [40], which primarily investigated its effects on parameters of left ventricular functions. The secondary indicators, dyspnea, and congestion biomarkers did not differ between groups. Contrary to other inotropes, no elevation in troponin has been seen with istaroxime use. Given the safety profile of istaroxime compared to other inotropes and its potential benefits in a subset of patients with hypotension and low cardiac output, it may be a viable option for high-risk groups with poor outcomes and a lack of evidence for safe and effective treatments. Further research into the effects of istaroxime on congestion is warranted in this population.

##### Cimlanod

STAND-UP AHF [41] was designed to show well-tolerated dosages of cimlanod in patients with AHF. The primary endpoint was clinically relevant hypotension; hence, STAND-UP AHF could be primarily considered a safety study. The secondary efficacy outcomes of STAND-UP AHF are intriguing. Reductions in NT-proBNP with cimlanod use propose a positive role in decongestion. The mechanism by which decongestion was achieved in STAND-UP AHF patients is unclear, although the transient nature of the changes in NT-proBNP and their resolution once the infusion was stopped favours volume redistribution over loss. The observation that cimlanod had no discernible effect on daily urine volumes supports this interpretation [62]. STAND-UP AHF results establish a safety baseline for future pivotal studies using primary indicators consistent with the drug’s potential mechanism of action, namely symptom relief and reduction in worsening HF episodes.

#### 4.2.2. Optimising Inotropy: Novel Myotrope Therapies

##### Omecamtiv Mecarbil

Omecamtiv mecarbil is a first-in-class cardiac myosin activator. Our review found one trial, ATOMIC-AHF [42], which examined the efficacy of omecamtiv in AHF patients. Patients were randomised to receive omecamtiv mecarbil or placebo in an ascending cohort design in this trial. The compiled data of the three cohorts of ATOMIC-AHF did not show dyspnea improvement. However, patients in the highest dose cohort experienced significantly increased dyspnea relief compared with those treated with placebo. It is reasonable to explain the positive role of omecamtiv by its pharmacological effects [20]. Earlier clinical studies showed that omecamtiv might provoke a risk of myocardial ischemia [20,42]; thus, ATOMIC-AHF used an intensive sampling of cardiac troponin in all patients. More patients randomised to omecamtiv mecarbil had elevated troponins than those allocated to placebo. Therefore, the regulatory, clinical program for omecamtiv mecarbil in AHF has been halted [20].

### 4.3. Novel Drug Targets in Clinical Development AHF—Sodium-Glucose Transporters (SGLT2 (Sodium-Glucose Cotransporter 2)) Inhibitors

In chronic settings, treatment with SGLT_2_ inhibitors reduced mortality and provided positive renal outcomes in patients with and without diabetes [5,14]. However, the clinical utility of SGLT_2_ inhibitors in acute settings is still lacking. Only one small study (EMPA—RESPONSE—AHF) [43] investigated the benefits of adjuvant use of empagliflozin. Despite the use of the osmotic diuresis and natriuresis associated with empagliflozin, decongestion efficacy parameters were considered comparable between both study groups. However, the results of this study must be interpreted with caution due to multiple serious limitations. First, the individual diuretic differences in the treatment of their patients due to the lack of a standardised diuretic protocol that guides initial dosing and titration of loop diuretic regimens. These prescribing variations might impact the results. Another notable limitation is the lack of stratified randomisation based on the presence or absence of diabetes mellitus, a critical confounder associated with diuretic resistance in AHF patients. Further, larger studies are highly warranted to examine the effects of higher doses and different options of SGLT2 inhibitors on improving decongestion in patients with AHF.

### 4.4. The Gap in Evidence and Implications of Future Research

Compelling evidence for new treatment options for chronic heart failure forced major changes in clinical practice guidelines for those patients. However, so far, the management of AHF still poses challenges and many areas with a lack of evidence persist. Table 4 displays selected vital issues that must be addressed in future clinical research to guide the synthesis of robust evidence-based recommendations.

### 4.5. Strengths and Limitations

This study, which utilised four major databases, sought to examine the current body of evidence regarding the efficacy of pharmacotherapy in treating congestion in patients with AHF. To the best of the authors’ knowledge, this is the first comprehensive systematic review that includes fourteen potential therapeutic interventions. A limitation of this review was the lack of meta-analysis and estimation of effect sizes, which was primarily due to the heterogeneity of the included studies. In addition, excluding studies published in languages other than English may have introduced a bias due to language. However, we tracked citations and manually searched all included studies to minimise the impact of factors (e.g., inconsistent terminology or improper indexing) that could affect the keyword-based search.

## 5. Conclusions

Based on the current evidence, clinicians may offer tolvaptan or serelaxin as an adjuvant therapy in AHF patients to tackle diuretic resistance. Rolofylline, neseritide, and omecamtiv mecarbil should not be used. The evidence for using SGLT2 inhibitors, cimlanod and levosimendan, is inconclusive for distinct reasons, including safety concerns, limited number of studies, or inconsistency between studies. This review, while comprehensive, is limited by the considerable variations in the primary outcome measures. Further studies with a larger sample size should focus on using a standardised protocolised diuretic treatment and measurement scales. The timing of treatment entry should also be considered in future studies.

## Figures and Tables

**Table 1:** Summary of studies investigated vasodilatory therapies.

Trial		Sample Size	Outcome(s)	Conclusion
Tolvaptan				
1	AQUAMARINE	Matsue et al., 2016 [24] Japan (*n* = 217)	Primary endpoint: urine output (UOP) within 48 h of hospitalisation. Secondary endpoints: Improvement of dyspnea from baseline measured on patient-reported 7-point Likert scale up to 48 h after enrollment. Change in B-type natriuretic peptide (BNP) Change in body weight	In AHF patients with renal dysfunction, adding tolvaptan to conventional therapy increased diuresis and alleviated dyspnea symptoms.
2	TACTICS-HF	Felker et al., 2017 [25] USA (*n* = 257)	Primary endpoint: The proportion of patients who improved at least moderately in dyspnea on a 7-point Likert scale after 8 and 24 h. Secondary endpoints: Dyspnea relief, fluid loss, change in body weight, the proportion of patients free from clinical congestion at 48 and 72 h	Tolvaptan did not improve the proportion of AHF patients classified as responders.
3	EVEREST	Gheorghiade, et al., 2007 [26] Multi. (*n* = 4133)	Primary endpoints: Composite score of changes from baseline in patient-assessed global clinical status and body weight.	Tolvaptan improved symptoms in AHF patients.
4	SECRET	Konstam et al., 2017 [27] USA (*n* = 250)	Primary endpoint: Change in dyspnea score (Likert scale). Secondary endpoints: Change in body weight. Other endpoints: Change in BNP	Tolvaptan showed improvement in dyspnea and weight loss.
5	K STAR	Inomata et al., 2017 [28] Japan (*n* = 81)	Primary endpoint: The average change in UOP compared with its baseline values. Secondary endpoints: Changes in body weight, and congestive signs and symptoms	Tolvaptan increased diuresis without further renal impairment.
6	ACTIV	Gheorghiade et al., 2004 [29] USA (*n* = 250)	Primary endpoints: Change in body weight at 24 h Worsening heart failure (WHF) Secondary endpoints: Changes in dyspnea, oedema, UOP, diuretics use, patient- and physician-assessed symptom scales.	Tolvaptan decreased bodyweight more effectively than standard therapy.
Conivaptan				
7		Goldsmith et al., 2008 [30] USA (*n* = 170)	Did not specify a primary endpoint Change in patient-assessed severity of dyspnea Change in global status (VAS score) UOP	Conivaptan safely improves UOP but does not relieve dyspnea.
Tezosentan				
8	VERITAS I, II	McMurray et al., 2007 [31] Multi. (*n* = 1435)	The primary endpoint of the individual studies: changes in dyspnea using a VAS over 24 h.	Tezosentan did not improve symptoms in AHF patients.
Serelaxin				
9	Pre-RELAX AHF	Teerlink et al., 2009 [32] Multi. (*n* = 234)	Primary endpoints (not prespecified): the overall effect of relaxin across several clinical domains: Relief of dyspnea (Likert scale and VAS). In-hospital WHF	Relaxin (30μg/kg) use relieved dyspnea.
10	RELAX-AHF	Teerlink et al., 2013 [33] Multi. (*n* = 1161)	Primary endpoints: Relief of dyspnea (Likert scale), and by VAS.	Treatment with serelaxin was associated with dyspnea relief.
Neseritide				
11	VMAC	Publication Committee for the VMAC Investigators, 2002 [34] (*n* = 489)	Primary endpoints: The absolute changes in PCWP The patient’s self-evaluation of dyspnea	Nesiritide improves hemodynamic function and dyspnea more effectively than placebo.
12	ASCEND-HF	O’Connor et al., 2011 [35] Multi. (*n* = 7141)	Primary endpoint: Co-primary endpoints of dyspnea change after six and 24 h (Likert scale).	Nesiritide has a nonsignificant effect on dyspnea.
13		Fu et al., 2012 [36] China (*n* = 140)	Primary endpoints not specified Dyspnea using the medical research council (MRC) scales. Assessment of oedema Assessment of water loss volume	Nesiritide was associated with better symptoms relief, such as dyspnea and oedema.
Rolofylline				
14	PROTECT pilot PROTECT pilot study	Cotter et al., 2008 [37] Multi. (*n* = 301)	Composite primary trichotomous endpoint: Patient-reported dyspnea (7-point Likert scale), WHF and worsening renal insufficiency. Patients were classified as improved, worse, or unchanged).	Rolofylline improved dyspnea relief and decreased worsening heart failure or renal function.
15	PROTECT	Massie et al., 2010 [38] Multi. (*n* = 2033)	The primary endpoint (clinical composite) Treatment success, i.e., moderate/marked improvement in dyspnea. Treatment failure, death or readmission for heart failure (HF) or worsening heart failure WHF No change in the patient’s condition.	Rolofylline does not show promise in treating patients AHF with renal dysfunction.

*n* = number of patients; Multi.: multinational; AHF: Acute heart failure; RCT: randomised controlled trial; UOP: urine output; BNP: Brain natriuretic peptide; PCWP: pulmonary capillary wedge pressure; VAS: visual analogue scale; NT-proBNP: NT-proB-type natriuretic peptide; WHF: worsening heart failure.

**Table 2:** Summary of studies investigated novel calcitrope and myotrope therapies.

Trial		Sample Size	Outcome(s)	Conclusion
Calcitrope trials				
Levosimendan				
16	REVIVE I and II	Packer et al., 2013 [39] USA Revive (I) (*n* = 100), (II) (*n* = 600)	Composite endpoint of clinically Patient-reported measures: Improved: moderate/markedly improvement Worse: Persistent/unresponsive symptoms Unchanged	levosimendan can produce significant symptomatic benefits.
Istaroxime				
17		Carubelli et al., 2020 [40] Multi. (*n* = 120)	Secondary endpoints Changes in dyspnea by VAS, changes in NT-proBNP, WHF	Istaroxime use did not add benefit to the diuretic response.
Cimlanod				
18	STAND-UP AHF	Felker et al., 2021 [41] Multi. (*n* = 322)	Secondary endpoints: Change in plasma concentration of NT-proBNP. Change in patient-reported resting dyspnea using the AUC of the numeric rating scale.	Cimlanod marginally improved some parameters related to congestion.
Myotrope trials: Omecamtiv mecarbil				
19	ATOMIC-AHF	Teerlink et al., 2016 [42] Multi. (*n* = 606)	Primary endpoint Dyspnea relief (Likert scale) Secondary endpoints Dyspnea numerical response AUC Patient global assessment response NT-proBNP change from baseline.	In patients with AHF, omecamtiv mecarbil had no significant effect on dyspnea.

*n*= number of patients; VAS: visual analogue scale; NT-proBNP: NT-proB-type natriuretic peptide; WHF: worsening heart failure; AUC: area under the curve.

**Table 3:** Summary of studies investigated miscellaneous therapies.

Trial		Sample Size	Outcome(s)	Conclusion
Empagliflozin				
20	EMPA-RESPONSE-AHF	Damman et al., 2020 [43] Netherlands (*n* = 80)	Primary endpoints Change in the AUC of dyspnea visual analogue scale Diuretic response Percentage change in NT-proBNP	Empagliflozin did not enhance diuretic response.
Thiamine				
21	Smithline et al., 2019 [44] Multi. (*n* = 118)		Primary endpoint Dyspnea severity using VAS in three positions: sitting upright on supplemental oxygen, sitting upright off oxygen, or lying supine off oxygen.	The results of this study do not support the adjuvant use of thiamine in AHF.
Clevidipine				
22	PRONTO	Peacock et al., 2014 [45] Multi. (*n* = 104)	Secondary endpoint: Dyspnea reduction (VAS score) at different time points up to 720 min after administration	Clevidipine effectively lowers blood pressure and improves dyspnea in hypertensive AHF patients.
Glucocorticoid				
23	COPE-ADHF	Liu et al., 2014 [46] (*n* = 102)	Other Outcomes Patient-assessed dyspnea (7-point scale). Physician-assessed global clinical (7-point scale).	This preliminary trial shows the potential benefit of short-term glucocorticoid use in patients with ADHF.

*n* = number of patients; AUC: area under the curve; VAS: visual analogue scale; NT-proBNP: NT-proB-type natriuretic peptide; ADHF: acute decompensated heart failure; AHF: acute heart failure.

**Table 4:** Common pitfalls in AHF studies and evidence-based practice recommendations for inpatient treatment of AHF.

Common Pitfalls in AHF Studies	Recommendations
**Lack of standardised algorithm for diuretic therapy**	Trials with primary efficacy indicators of diuresis require a protocolised algorithm that guides dose adjustment based on response (Na in urine and UOP) to reduce the impact of prescribing variations on the diuretic therapy results. The most recent European society of cardiology practice guidelines [5] and ongoing clinical trials have endorsed a similar approach [18].
**Heterogeneity in efficacy indicators impeded the evaluation of potential therapies:** Variations in the applied dyspnea scales. Diversity in the timing of measurement	Variability could be minimised [19] via: Consensus-building on measurement tools that use standardised unidimensional scales and timings. Using a standardised operation and protocols to assess the clinical efficacy of potential dyspnea therapies. The use of AUC to quantify relief in symptoms measured by the VAS scale at different time points might help standardise the comparison between different options. A similar approach was followed in different clinical situations that utilized the VAS scale as an outcome measure [63], including dyspnea assessment in AHF [32,33,43].
**Time-to-treatment [5]**	As with acute coronary syndrome, current guidelines advocate a ‘time-to-treatment’ concept and recommend early treatment in patients with AHF, ideally prior to hospital admission.

## Data Availability

Not applicable.

## Supplementary Materials

The following supporting information can be downloaded at: https://www.mdpi.com/article/10.3390/jcm11113112/s1. The Supplementary File includes detailed summary of the included studies.
