# Supplementary material for: Efficacy of Nondiuretic Pharmacotherapy for Improving the Treatment of Congestion in Patients with Acute Heart Failure: A Systematic Review of Randomised Controlled Trials
Source: J Clin Med. 2022 May 31;11(11):3112. doi: 10.3390/jcm11113112 (PMC9181246; doi:10.3390/jcm11113112)
Supplement: Supplementary file 1 [file jcm-11-03112-s001.zip › jcm-1654638-supplementary.pdf]

Trial			Study design, Arms, and Objectives	Outcomes	Results	Conclusion
**Vasodilatory Therapies**						
**Vasopressin Receptor Antagonists**						
**Tolvaptan (N= 6)**						
1	AQUAMARINE	Matsue et al., 2016[23] Japan	RCT (n=217 AHF and renal dysfunction) compared the efficacy of adding tolvaptan 15 (mg/day) to loop diuretics	Primary endpoint: UOP within 48h of hospitalisation.<br>Secondary endpoints:<br>- Improvement of dyspnea from baseline measured on patient-reported 7-point Likert scale up to 48 h after enrollment.<br>- Change in B-type natriuretic peptide (BNP)<br>- Change in body weight	- The tolvaptan enhanced diuresis more than the conventional treatment (6464.4 vs 4999.2 mL; P < .001)<br>- At all time points within 48 hours, except 6 hours after randomisation, dyspnea improved significantly in the tolvaptan group than in the placebo group.<br>- Significantly more significant weight loss in the tolvaptan group<br>- No significant difference in absolute BNP reduction.	In AHF patients with renal dysfunction, adding tolvaptan to conventional therapy increased diuresis and alleviated dyspnea symptoms.
2	TACTICS-HF	Felker et al., 2017[30] USA	RCT (n=257 patients with AHF) received either 30 mg of oral tolvaptan or a placebo	Primary endpoint: The proportion of patients who improved at least moderately in dyspnea on a 7-point Likert scale after 8 and 24 hours.<br>Secondary endpoints:<br>- Dyspnea relief<br>- Fluid loss<br>- Change in body weight<br>- The proportion of patients free from clinical congestion at 48 and 72 h	- Proportion of patients experienced dyspnea relief did not differ significantly between tolvaptan and placebo groups at 8 h (p = 0.59) and at 24 h (p = 0.80).<br>- For the 48-hour treatment period, Tolvaptan was linked to significantly greater weight and fluid loss.	Tolvaptan did not improve the proportion of AHF patients classified as responders.

3	EVEREST	Gheorghiade, et al.,2007[31] Multinational	RCT (n=4133 patients with AHF) assessed the effects of tolvaptan 30 mg/day versus placebo.	Primary endpoints:<br>- Composite score of changes from baseline in patient-assessed global clinical status and body weight.	- Tolvaptan showed superiority about the composite endpoint at day 7, P<0.001 and the bodyweight reduction at day one and day 7: P<0.001.<br>- Global clinical status improvements were comparable between groups.	Tolvaptan improved symptoms in AHF patients.
4	SECRET	Konstam et al., 2017[24] USA	RCT (n=250 patients with AHF) assessed the effects of tolvaptan 30 mg/day versus placebo.	Primary endpoint:<br>- Change in dyspnea score (Likert scale).<br>Secondary endpoints:<br>- Change in body weight.<br>Other endpoints:<br>- Change in BNP	- At day 3, tolvaptan was associated with significant dyspnea reduction (81.2% vs. 66.3%; p = 0.02) and greater weight loss (p < 0.01), than the placebo group.<br>- There were no reported differences in BNP levels between study groups.	Tolvaptan showed improvement in dyspnea and weight loss.
5	K STAR	Inomata et al., 2017[32] Japan	RCT in 81 patients with fluid retention (despite taking ⩾40 mg/day furosemide, with an eGFR <45 mL/min/1.73m2 administered either ⩽15 mg/day tolvaptan or ⩽40 mg/day increased furosemide.	Primary endpoint:<br>- The average change in UOP compared with its baseline values.<br><br>Secondary endpoints:<br>- Changes in body weight<br>- Congestive signs and symptoms	- Urine volumes were significantly higher in the tolvaptan than the furosemide group.<br>- There was a modest difference in body weight or improved congestion between the two groups	Tolvaptan increased diuresis without further renal impairment.
6	ACTIV	Gheorghiade et al., 2004 [25] USA	RCT (n=250 patients) compared different doses (30, 60, or 90 mg/d) of tolvaptan versus placebo in addition to standard therapy	Primary endpoints:<br>- Change in body weight at 24 h<br>- worsening heart failure<br><br>Secondary endpoints:<br>- Changes in dyspnea, oedema, urine output, diuretics use, patient- and physician-assessed symptom scales.	- At 24 h, body weight decreased in tolvaptan groups 30, 60, and 90 mg/d compared to placebo (P< or =.008).<br>- No significant difference in worsening heart failure was reported between tolvaptan and placebo groups.<br>- Differences in secondary endpoints were not significant except for dyspnea.	Tolvaptan decreased bodyweight more effectively than standard therapy.
**Conivaptan**						

7		Goldsmith et al., 2008[33] USA	RCT (n=170 patients) dose-finding study; examined different escalating doses of conivaptan or placebo.	Did not specify a primary endpoint<br>- Change in patient-assessed severity of dyspnea<br>- Change in global status (VAS score)<br>- Urinary output	- Both groups were comparable in both patient or clinician global and respiratory status assessments at 48 h.<br>- At 24 hours, conivaptan increased UOP significantly more than placebo (P=0.02).	Conivaptan safely improves UOP but does not relieve dyspnea.
**Tezosentan**						
8	VERITAS I, II	McMurray et al., 2007[34] Multi	RCT (n=1435 patients with AHF) evaluated the effect of adjuvant tezosentan use.	The primary endpoint of the individual studies:<br>- changes in dyspnea using a VAS over 24 h.	- Tezosentan did not improve dyspnea more than placebo	Tezosentan did not improve symptoms in AHF patients.
**Serelaxin (N= 2)**						
9	Pre-RELAX AHF	Teerlink et al., 2009 [26] Multinational	RCT (n= 234) dose-finding study compared different relaxin doses 10, 30, 100, or 250 µg/kg vs placebo	Primary endpoints (not prespecified) :the overall effect of relaxin across several clinical domains:<br>- Relief of dyspnea (Likert scale and VAS).<br>- In-hospital worsening heart failure	- Absolute increase in early and sustained dyspnea relief was found in 17·5%, vs 76% in placebo and relaxin (30 µg/kg), respectively.<br>- The VAS changes proposed a positive effect on dyspnea relief.	Relaxin (30µg/kg) use relieving dyspnea.
10	RELAX-AHF	Teerlink et al., 2013 [27] Multinational	RCT (n=1161 patients with AHF) receive either 30 µg/kg/day serelaxin or placebo	Primary endpoints:<br>- Relief of dyspnea (Likert scale), and by VAS.	- Serelaxin improved the VAS AUC dyspnea endpoint (448 mm × h; p=0·007) compared with placebo but had no significant effect on the other primary outcomes	Treatment with serelaxin was associated with dyspnea relief.
**Neseritide (N= 3)**						

11	VMAC	Publication Committee for the VMAC Investigators, 2002 [35]	RCT (n= 489 patients) compared the efficacy of nesiritide, nitroglycerin, and placebo.	Primary endpoints:<br>- The absolute changes in PCWP<br>- The patient's self-evaluation of dyspnea	- Nesiritide decreased PCWP from baseline more than placebo (P<0.001) and improved early dyspnea compared with placebo (P=.03).	Nesiritide improves hemodynamic function and dyspnea more effectively than placebo.
12	ASCEND-HF	O'Connor et al., 2011 [36] Multinational	RCT (n= 7141 patients with AHF compared nesiritide or placebo	Primary endpoint:<br>Coprimary endpoints of dyspnea change after six and 24hr (Likert scale).	- Nesiritide showed non-significant improvements in dyspnea at 6 h (44.5% vs. 42.1%) and 24 h (68.2% vs. 66.1%), compared to placebo.	Nesiritide has a nonsignificant effect on dyspnea.
13		Shihui Fu et al., 2012 [37] China	RCT (n= 140 geriatric patients) compared conventional treatment vs nesiritide.	Primary endpoints not specified<br>- Dyspnea using the medical research council (MRC) scales.<br>- Assessment of oedema<br>- Assessment of water loss volume	- The nesiritide group had significantly lower MRC scales (p<0.05) and more net body fluid losses (p<0.05) compared to the control group<br>- Sores of oedema had no significant difference on day four between the two groups (p>0.05) but were significantly lower in the nesiritide group on days 8 and 14 (all p<0.05).	Nesiritide was associated with better symptoms relief, such as dyspnea and oedema.
**Rolofylline (N=2)**						

14	PROTECT pilot study	Cotter et al., 2008 [38] Multinational	RCT (n=301 patients with AHF and renal impairment) dose-finding study compared placebo or rolofylline 10, 20, or 30 mg.	Composite primary trichotomous endpoint:<br>- Patient-reported dyspnea (7-point Likert scale), worsening heart failure and worsening renal insufficiency.<br>- Patients were classified as improved, worse, or unchanged).	- Patient-reported dyspnea: rolofylline significantly improved dyspnea compared to placebo (52.7% vs 37.2%).<br>- Comparing rolofylline 30 mg (the dose selected for the pivotal study) with placebo, the estimated odds ratio was 0.51 (95% CI 0.28-0.94) for the Composite primary trichotomous endpoint.	Rolofylline improved dyspnea relief and decreased worsening heart failure or renal function.
15	PROTECT	Massie et al., 2011 [39] Multinational	RCT (n=2,033) patients with dyspnea at rest or minimal activity, estimated CrCl 20–80 mL/ min, BNP ≥500 pg/mL to rolofylline 30 mg/day or placebo for up to 3 days).	The primary endpoint (clinical composite)<br>- Treatment success, i.e., moderate /marked improvement in dyspnea.<br>- Treatment failure, death or readmission for heart failure (HF) or worsening heart failure (WHF)<br>- No change in the patient's condition.	- Rolofylline was not found to be superior to placebo in terms of the primary endpoint (odds ratio, 0.92; P=0.35).	Rolofylline does not show promise in treating patients AHF with renal dysfunction.
**Calcitrope trials**						
**Levosimendan**						
16	REVIVE I and II	Packer et al., 2013 [40] USA	Revive I) n =100), II) n=600)patients with ADHF) evaluated the effect of adjuvant levosimendan use.	Composite endpoint of clinically Patient-reported measures:<br>- Improved: moderate / markedly improvement<br>- Worse: Persistent /unresponsive symptoms<br>- Unchanged	- More patients were improved in the levosimendan group compared to placebo (58 vs 44).<br>- Clinical worsening occurred in fewer levosimendan patients (58 vs 82, p = 0.015).	levosimendan can produce significant symptomatic benefits.
**Istaroxime**						

17		Carubelli et al., 2020 [41] Multinational	RCT (n=120 AHF patients with reduced LVEF and low SBP) evaluated the efficacy of two different doses of istaroxime (0.5 and 1.0 µg/kg/min) compared to placebo.	Secondary endpoints<br>- Changes in dyspnea by VAS<br>- Changes in NT-proBNP<br>- Worsening heart failure	- There were no significant differences between istaroxime and placebo on any secondary endpoints.<br>- Overall diuresis was numerically more significant in istaroxime-treated patients during a 24-hour infusion.	Istaroxime use did not add benefit to the diuretic response.
**Cimlanod**						
18	STAND-UP AHF	Felker et al., 2021[42] Multinational	RCT (n=322 patients) done in 2 sequential cohorts<br>- Cohort I: escalate cimlanod doses or placebo.<br>- Cohorts II: use the highest tolerated doses of cimlanod (6 and 12 mg/kg/min) or placebo.	Secondary endpoints:<br>- Change in plasma concentration of NT-proBNP.<br>- Change in patient-reported resting dyspnea using the AUC of the numeric rating scale.	- The reduction in NT-proBNP was more significant with cimlanod than with placebo.<br>- Dyspnea symptoms were generally comparable between patients assigned to cimlanod and placebo.	Cimlanod marginally improved some parameters related to congestion.
**Myotrope trials**						
**Omecamtiv mecarbil**						
19	ATOMIC-AHF	Teerlink et al., 2016[43] Multinational	RCT (n=606 patients), a dose-finding study, evaluated the effect of omecamtiv mecarbil on dyspnea relief. Patients were randomised in three sequential cohorts to escalate doses (low, medium or high) versus placebo.	Primary endpoint<br>- Dyspnea relief (Likert scale)<br><br>Secondary endpoints<br>- Dyspnea numerical response AUC<br>- Patient global assessment response<br>- NT-proBNP change from baseline.	- Dyspnea relief with omecamtiv mecarbil marginally differed from pooled placebo of the three doses cohorts ($p=0.33$).<br>- There were no statistically significant differences in secondary endpoints between treatment cohorts and the pooled placebo.<br>- The high dose cohort had more remarkable dyspnea improvement than its matching placebo group ($p = 0.038$).	In patients with AHF, omecamtiv mecarbil had no significant effect on dyspnea.
**SGLT2 Inhibitors**						
**Empagliflozin**						

20	EMPA-RESPONSE-AHF	Damman et al 2019[29] Netherlands	RCT (n= 80 patients) with AHF, diabetic and non-diabetic. Patients were randomised to 10mg/day empagliflozin or placebo as an adjuvant to loop diuretics.	Primary endpoints<br>- Change in the AUC of dyspnea visual analogue scale<br>- Diuretic response<br>- Percentage change in NT-proBNP	- No difference was seen in the AUC of dyspnea VAS over the first four days (P = 0.18) in the empagliflozin and placebo groups, respectively.<br>- Diuretic response and percentage change in NT-proBNP was comparable in empagliflozin and placebo group, (P = 0.37, 0.63, respectively).	Empagliflozin did not enhance diuretic response.
**Miscellaneous**						
**Thiamine**						
21	Smithline et al., 2019 [44] Multinational		RCT (n= 118 patients) examined the effect of adding thiamine to the standard of care	Primary endpoint<br>- Dyspnea severity using VAS in three positions: sitting upright on supplemental oxygen, sitting upright off oxygen, or lying supine off oxygen.	- There was only one significant difference between groups over time in dyspnea measured sitting upright on oxygen (P=0.015). Dyspnea did not change in any of the other positions.	The results of this study do not support the adjuvant use of thiamine in AHF.
**Clevidipine**						
22	PRONTO	Peacock et al., 2014[45] Multinational	RCT to study the efficacy of Clevidipine vs standard of care in patients (n=104) with HF having SBP ≥160 mmHg and dyspnea ≥50 on a scale (VAS)	Secondary endpoint:<br>- Dyspnea reduction (VAS score) at different time points up to 720 minutes after administration	- Clevidipine patients had more remarkable mean VAS dyspnea improvement than standard care (-37 vs -28mm, p=0.02) at 45 minutes, a difference that persisted for up to 3 h.	Clevidipine effectively lowers blood pressure and improves dyspnea in hypertensive AHF patients.
**Glucocorticoid**						

23	COPE-ADHF	Liu et al., 2014[46]	RCT (n=102 patients) to assess the efficacy of glucocorticoid therapy (single dose dexamethasone followed by prednisolone daily for 1week).	Other Outcomes<br>- Patient-assessed dyspnea (7-point scale).<br>- Physician-assessed global clinical (7-point scale).	- After seven days, the glucocorticoid-treated group had greater patient-assessed dyspnea improvement than the standard treatment group (2.71 vs 1.78 points, P < 0.01).<br>- Improvement in physician-assessed global clinical status, favouring glucocorticoids, were also found (P < 0.01).	This preliminary trial shows the potential benefit of short-term use of glucocorticoid in patients with ADHF.

AHF: Acute heart failure; RCT: randomised controlled trial; UOP: urine output; BNP: Brain natriuretic peptide; PCWP: pulmonary capillary wedge pressure; VAS: visual analogue scale; AUC: area under the curve; ADHF: Acute decompensated heart failure; SBP: systolic blood pressure; NT-proBNP: NT-proB-type natriuretic peptide; LVEF: Left ventricular ejection fraction (LVEF); WHF: worsening heart failure; CrCl creatinine clearance.
